# Influence of MRI-based boundary conditions on type B aortic dissection simulations in false lumen with or without abdominal aorta involvement

**DOI:** 10.3389/fphys.2022.977275

**Published:** 2022-09-07

**Authors:** Dongting Liu, Xuan Wang, Dongliang Zhao, Zhonghua Sun, Jumatay Biekan, Zhaoying Wen, Lei Xu, Jiayi Liu

**Affiliations:** ^1^ Department of Radiology, Beijing Anzhen Hospital, Capital Medical University, Beijing, China; ^2^ Department of Mechanics and Engineering Science, College of Engineering, Peking University, Beijing, China; ^3^ Discipline of Medical Radiation Science, Curtin Medical School, Curtin University, Perth, WA, Australia; ^4^ Circle Cardiovascular Imaging, Calgary, AB, Canada

**Keywords:** type B aortic dissection, computed tomography angiography, computational fluid dynamics, 4D flow MRI, modeling

## Abstract

Most computational hemodynamic studies of aortic dissections rely on idealized or general boundary conditions. However, numerical simulations that ignore the characteristics of the abdominal branch arteries may not be conducive to accurately observing the hemodynamic changes below the branch arteries. In the present study, two men (M-I and M-II) with type B aortic dissection (TBAD) underwent arterial-phase computed tomography angiography and four-dimensional flow magnetic resonance imaging (MRI) before and after thoracic endovascular aortic repair (TEVAR). The finite element method was used to simulate the computational fluid dynamic parameters of TBAD [false lumen (FL) with or without visceral artery involvement] under MRI-specific and three idealized boundary conditions in one cardiac cycle. Compared to the results of zero pressure and outflow boundary conditions, the simulations with MRI boundary conditions were closer to the initial MRI data. The pressure difference between true lumen and FL after TEVAR under the other three boundary conditions was lower than that of the MRI-specific results. The results of the outflow boundary conditions could not characterize the effect of the increased wall pressure near the left renal artery caused by the impact of Tear-1, which raised concerns about the distal organ and limb perfused by FL. After TEVAR, the flow velocity and wall pressure in the FL and the distribution areas of high time average wall shear stress and oscillating shear index were reduced. The difference between the calculation results for different boundary conditions was lower in M-II, wherein FL did not involve the abdominal aorta branches than in M-I. The boundary conditions of the abdominal branch arteries from MRI data might be valuable in elucidating the hemodynamic changes of the descending aorta in TBAD patients before and after treatment, especially those with FL involving the branch arteries.

## Introduction

Aortic dissection is caused by a tear in the inner layer of the aortic wall, allowing blood to enter a new channel (false lumen, FL) and separating the middle layer to create two channels [true lumen (TL) and FL] (Pretre and VonSegesser, 1997; Golledge and Eagle, 2008). This phenomenon might be related to intimal thickening caused by diseases, such as hypertension and atherosclerosis, which affect the progression of aortic dissection (Nienaber et al., 2016). According to the position of the primary entry, the aortic dissection is classified into Stanford and DeBakey types. Stanford type A aortic dissection involving the ascending aorta necessitates urgent surgical treatment. Conversely, Stanford type B aortic dissection involving the descending aorta is often managed with medication or surgical or endovascular intervention in case of complications (Hiratzka et al., 2010; Afifi et al., 2015). Thoracic endovascular aortic repair (TEVAR) is an essential tool for the treatment of type B aortic dissection (TBAD), and the core principle of TEVAR involves placing a covered stent graft over the entry tear of the descending aorta (Dillon-Murphy et al., 2016).

Several studies have used finite element calculation to discern the complex phenomena underlying the development of TBAD (Wang et al., 2017; Wang et al., 2018). The characterization of hemodynamic parameters and the analysis of vascular morphological features are essential for understanding the pathogenesis of vascular disease. Some studies have shown that pressure is a significant predictor of disease prognosis, and wall shear stress (WSS) is a critical driving force for aortic remodeling (Davies, 2009; Menichini et al., 2016), which can strongly affect the formation and progression of aortic dissection (Chen et al., 2016). While exploiting computational fluid dynamics (CFD) to discern the correlation between the morphological characteristics of aortic dissection and hemodynamic parameters, some studies used computed tomography angiography (CTA) images to reconstruct the patient-specific geometry for enhanced analysis (Yin et al., 2016; Pirola et al., 2019). Presently, magnetic resonance imaging (MRI) is used clinically for the diagnosis of patients with aortic dissection in combination with CTA images. MRI also provides patient-specific velocity data (Auricchio et al., 2014) that can be applied as boundary conditions for CFD calculations, especially in the subacute and chronic stages of aortic dissection (Nienaber et al., 2016). Four-dimensional (4D) flow MRI is a 3D phase-contrast MRI equipped with time resolution (Allen et al., 2019), which can be used to collect variable velocity data in multiple directions to obtain complex 3D dynamic parameters. Subsequently, these parameters could be utilized to calculate various parameters related to intracranial and abdominal arterial blood flow.

Many studies have used typical flow waveforms to map onto flat or parabolic profiles at the inlet. However, idealized velocity profiles are not suitable for studies focusing on the ascending aorta and the aortic arch (Pirola et al., 2018; Pirola et al., 2019) because the descending aorta is less sensitive to the inlet velocity profile. Previous computational studies have included the hemodynamic characteristics of TBAD (Alimohammadi et al., 2014; Xu et al., 2017), flow effects after TEVAR (Karmonik et al., 2011a), impact of tear on blood flow (Karmonik et al., 2011b), and fluid-structure interaction (Alimohammadi et al., 2015). Due to the lack of patient-specific flow and pressure measurements, many studies have used simplified outlet boundary conditions, such as constant pressure (Polanczyk et al., 2018), resistance boundary conditions (Vignon-Clementel et al., 2006), or representative pressure waveforms. The flow through the primary entry tear enters the wall layers to split along the abdominal aorta. Moreover, the FL of some patients involves the visceral arteries in the abdomen that might influence organ perfusion, while in others, the visceral arteries are not involved.

Typically, the geometric models of the aorta and aortic dissection are established based on the descending aorta involving the superior mesenteric artery, the celiac artery, and the renal arteries. Furthermore, most models of aortic dissection, irrespective of FL, ignored the boundary conditions of the branch arteries in the abdomen (Alimohammadi et al., 2014; Alimohammadi et al., 2015; Bonfanti et al., 2017; Menichini et al., 2018; Xu et al., 2018), which might have a substantial impact on the flow in the FL. Boundary conditions refer to the mathematical and physical conditions that need to be satisfied by the flow field variables on the computational boundary. The solution of the flow field is accessible after initialization and is unique for a specific boundary condition. The selection of boundary conditions directly affect the accuracy of the calculated results. Pirola et al. (Pirola et al., 2019) compared the pre-stent grafting-MRI data with CFD results based on the patient’s data while the post-MRI data were not included. Currently, the hemodynamic analysis of abdominal aortic data based on patient-specific 4D flow MRI combined with CFD calculations is lacking (Qiao et al., 2015).

Thus, the present study aimed to investigate the time-flow curves of the aorta and the major branch arteries before and after TEVAR measured by 4D flow MRI data. Then, we compared the hemodynamics of the two types of TBAD under different boundary conditions of the abdominal arterial branches via numerical simulations: 1) Setting 1: boundary conditions based on 4D flow MRI data; 2) Setting 2: resistance boundary conditions; 3) Setting 3: zero pressure boundary conditions; 4) Setting 4: outflow boundary conditions. Herein, we aimed to observe the flow field, wall pressure, the time-average wall shear stress (TAWSS), and the oscillating shear index (OSI) of the abdominal aorta with boundary conditions based on MRI data for patients with and without the false lumen involving the visceral branches of the abdomen. Despite similar previous studies, the results of numerical simulations were compared by incorporating the features of the abdominal branch arteries into 4D flow MRI data to elucidate its importance in the analysis of hemodynamic changes in patients with aortic dissection.

## Methods

### Data acquisition and geometry reconstruction

The Institutional Review Board of the Beijing Anzhen Hospital Ethics Committee approved the study and written informed consent was obtained from the patients. Two male subjects with TBAD disease (Model-I: M-I and Model-II: M-II; 51- and 50-years-old; 72.6 and 78.4 kg, respectively) had a history of hypertension (180/110 mmHg and 160/100 mmHg, respectively). In addition, they were neither allergic to the contrast medium nor had a history of impaired heart, liver, or kidneys functions. They underwent aorta computed tomography angiography (CTA) on a 320-row CT scanner (Aquilion One, Canon Medical Systems, Japan) and 4D flow MRI scans on a 3 T MR system (MAGNETOM Vero, Siemens Healthcare, Erlangen, Germany), prior to TEVAR (Liu et al., 2018). The average heart rate for M-I and M-II was 73 and 76 beats/min, respectively. The FL of M-I, but not M-II, involved the visceral arteries in the abdomen. After TEVAR, thrombus formation was observed in the FL, which extended up to the celiac artery. FL thrombosis can help restore the blood flow in the TL after surgery (Menichini et al., 2018). A follow-up CTA and 4D flow MRI scan were performed 10 days post-TEVAR. The image segmentation and reconstruction of the aortic dissection based on CTA images were carried out using Mimics software (Materialise, Belgium).

**FIGURE 1:**
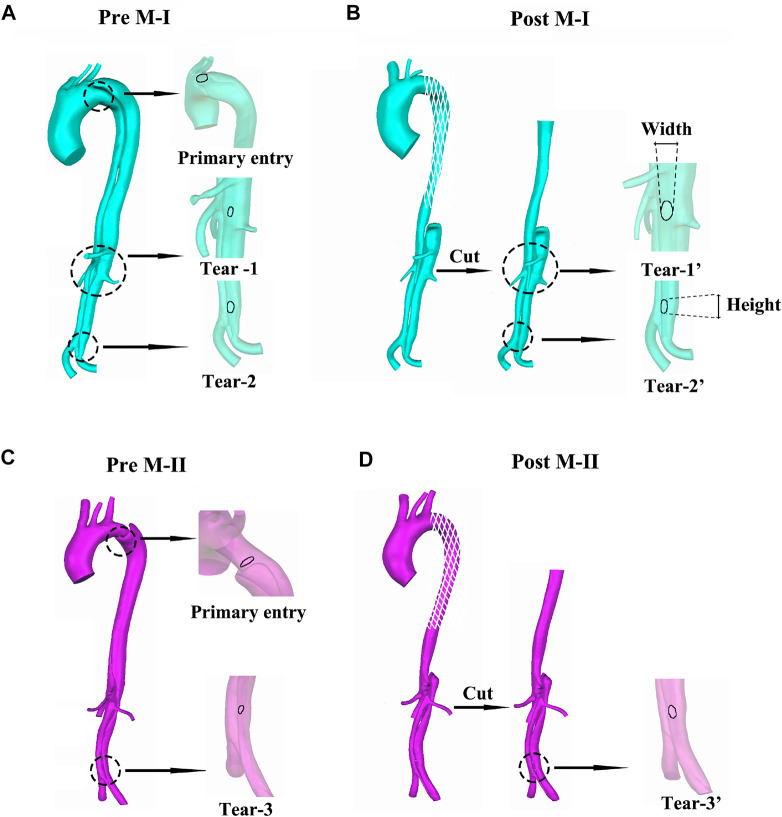
**(A–B)** display the preoperative (pre) and postoperative (post) reconstructed models of aortic dissection on M-I, and **(C–D)** on M-II.

**FIGURE 2:**
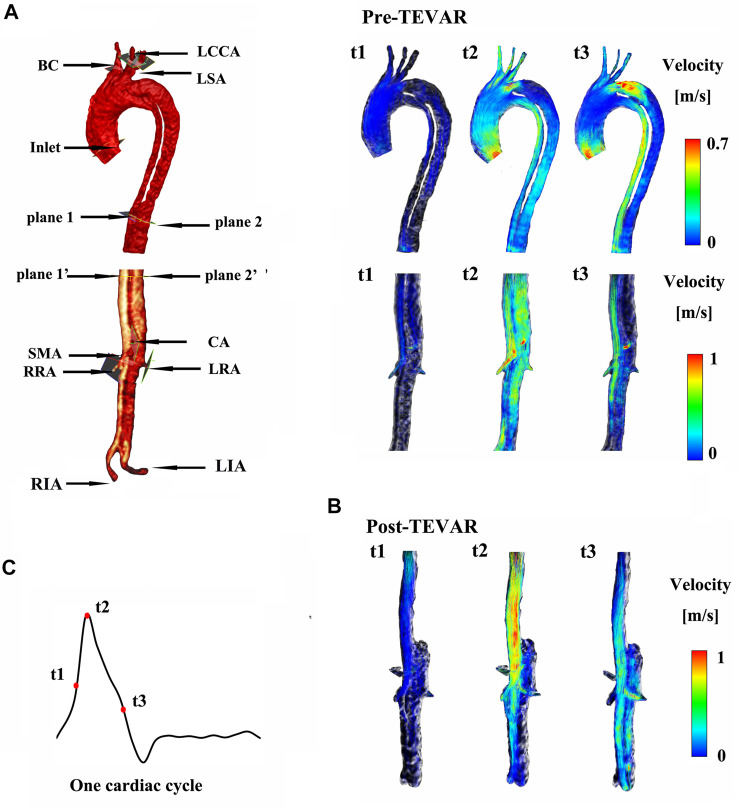
**(A)** shows the instantaneous streamlines of 4D MRI at three systolic time-points (t1–-t3) of M-I before thoracic endovascular aortic repair (pre-TEVAR), and **(B)** after TEVAR (post-TEVAR). **(C)** Shows three systolic time-points of one cardiac cycle on M-I.

As shown in Figure 1, the models included one inlet in the ascending aorta (Inlet) and nine outlets, including brachiocephalic artery (BC), left common carotid artery (LCCA), left subclavian artery (LSA), celiac artery (CA), superior mesenteric artery (SMA), left and right renal arteries (LRA and RRA), and left and right iliac arteries (LIA and RIA). Three aortic tears were detected in M-I before the surgery: the primary entry located at the proximal end of the descending aorta and the other two tears (Tear 1 and Tear 2). After surgery, the primary entry was sealed. However, only two aortic tears were detected in M-II before the surgery: the primary entry (sealed after the surgery) and Tear 3. The geometric outline of the reconstructed 3D model was consistent with the features of the CTA images (Xu et al., 2017). The pre-TEVAR images consisted of two parts (thoracic aorta and abdominal aorta), and the post-TEVAR images included only the abdominal aorta, as shown in Figure 2. The models were meshed with tetrahedral elements in the core area and prismatic cells (five layers) in the boundary layer near the aortic wall using Integrated Circuit Electromagnetic Model (ANSYS Inc, Canonsburg, United States). The grid resolution varied between 2,500,000 and 3,000,000 units.

### MRI processing and setting of the boundary conditions

4D flow MRI data obtained using prospective electrocardiogram and respiratory navigator gating (Allen et al., 2019) were recorded to calculate the specific boundary conditions for the pre- and post-TEVAR models. Pulse sequence parameters were as follows: temporal resolution = 47.2 ms, TE/TR = 2.79/5.90 ms, FA = 8°, FOV = (255 × 340) mm^2^, matrix = 115 × 192, slice thickness = 1.80 mm, BW = 491 Hz/px, GRAPPA acceleration factor = 2, and approximate imaging time = 6–9 min (Liu et al., 2018). Retrospective (respiratory) cardiac gating was used to obtain approximately 10–15 time points for each cardiac cycle, but no aliasing was observed. During CTA reconstruction, only the descending aorta was reconstructed after TEVAR. Moreover, the cvi42 prototype 4D flow module (cmr^42^, Circle Cardiovascular Imaging Inc, Calgary, Canada) was used to visualize the 4D flow MRI data (Childs et al., 2011) for analyzing the time-variant flow rate and diameter.

Time-variant velocities at the inlet and the three supra-aortic branches, BC, LCCA, and LSA, were calculated using the time-variant flow rate and the diameter-based 4D flow MRI data. Setting 3 or Setting 4 were set at the other outlets. Setting 3 is to apply zero pressure on the extended outlet, and then take the pressure value at the original outlet as the boundary condition to recalculate. Setting 4 assumes that the flow is fully developed in the direction perpendicular to the boundary surface. Fluent software uses the internal variables of the flow field to obtain the variable values on the outflow boundary conditions through interpolation. The branches of the abdominal aorta (CA, SMA, LRA, and RRA) were set with Setting 1 or Setting 2. Setting 1 is to apply time-variant velocity, and Setting 2 is to assume that resistance is defined as a constant relationship between average pressure and flow at each outlet of arterial tree according to the law of diameter flow (e.g. Supplementary File 1) from our previous study (Yin et al., 2016; Huang et al., 2018). The outlet pressure at the LIA and RIA was obtained from a previous study (Vignon-Clementel et al., 2006) (Table 1). All the velocity boundary conditions were calculated based on the assumed parabolic flow profile for the inlet and outlet sections. The vessel wall was rigid and a no-slip because of low distensibility in the long-term follow-ups (Ganten et al., 2009; Selene et al., 2019).

**TABLE 1:** Boundary condition setting for inlet and outlets.

	—	Setting 1	Setting 2	Setting 3	Setting 4
Pre/Post-Inlet	—	Patient specific velocity			
Pre/Post-Outlets	BC	Patient specific velocity			
—	LCCA	Patient specific velocity			
—	LSA	Patient specific velocity			
—	CA	Velocity	Resistance BC	Zero pressure	Outflow
—	SMA	Velocity	Resistance BC	Zero pressure	Outflow
—	LRA	Velocity	Resistance BC	Zero pressure	Outflow
—	RRA	Velocity	Resistance BC	Zero pressure	Outflow
—	LIA	Outlet pressure	Outlet pressure	Zero pressure	Outflow
—	RIA	Outlet pressure	Outlet pressure	Zero pressure	Outflow

### Numerical simulations

Blood is modeled as a type of incompressible Newtonian fluid (Dai et al., 2020), and the viscosity (μ) and density (ρ) were estimated as 3.5 × 10^–3^ Pa s and 1060 kg/m^3^, respectively. Based on clinical applicability, the use of laminar flow for Navier–Stokes equation is time- and computational cost-effective (Gallo et al., 2012; Alimohammadi et al., 2014; Xu et al., 2017; Yin et al., 2018; Manchester et al., 2022). The laminar flow simulation with a sufficiently small grid resolution, especially the refined simulation of the part close to the wall, can refine the flow disturbances occurring near the wall (Chen et al., 2013; Alimohammadi et al., 2014; Manchester et al., 2022). The SIMPLE-type pressure correction was used for pressure–velocity coupling, a second-order advection scheme was adopted for spatial discretization of the Navier–Stokes equation, and a second-order implicit backward Euler scheme was employed for temporal discretization (Pirola et al., 2019). The time-step and maximum RMS residual were set as 0.2 × 10^−3^ s and 1.0 × 10^−4^, respectively. To ensure developed velocity profiles at the inlet and minimize the influence of outlet boundary conditions, straight flow extensions with a length of twice the diameter were added to the inlet and outlet faces of the model (Gallo et al., 2012; Morbiducci et al., 2013; Auricchio et al., 2014).

Mesh dependency, skewness, and orthogonal quality metrics were analyzed to satisfy the quality of the grids, as described previously (Chen et al., 2016; Yin et al., 2018). The models were meshed in ICEM (ANSYS Inc, Canonsburg, United States) with tetrahedral elements in the core region and prismatic cells (10 layers) in the boundary layer near the aortic wall. The grid resolution varies from 3,500,000 to 4,000,000 cells; and the base time steps are 0.02 s respectively. Sensitivity analysis was carried out to assure a grid-independent solution (Supplementary Figure S1). To compare the results, a point near the proximal tear in the aortic arch of the TL has been studied (Supplementary Figure S1). The exact same trend of velocity magnitude change of Mesh 3 and Mesh 4 is observed. A simulation was performed for three cardiac cycles to obtain a periodic solution, and the results of the final cycle were presented.

The continuity and Navier–Stokes equations are as follows:

$$\nabla \cdot \vec{V}=0$$ (1)

$$\rho \frac{\partial \vec{V}}{\partial t}+\rho \vec{V}\cdot \nabla \vec{V}=-\nabla P+\nabla \cdot \mu \left(\nabla \vec{V}+{\left(\nabla \vec{V}\right)}^{T}\right)$$ (2)

Where, $\vec{V}=u{\hat{e}}_{x}++v{\hat{e}}_{y}+w{\hat{e}}_{z}$ ; ρ, *p*, and *μ* represent the velocity, density, pressure, and viscosity of the blood flow, respectively. The Navier–Stokes and continuity equations were used to determine the blood flow patterns using FLUENT; especially, the solutions to velocities can become periodic after three cardiac cycles (Romarowski et al., 2018; Pirola et al., 2019).

Hemodynamic Measurements: Similar to previous studies (Fan et al., 2016; Xu et al., 2017), the shear component of $\vec{\tau }$ (Supplementary File S1) was expressed as follows:

$${\vec{\tau }}_{shear}=\vec{\tau }-\left(\vec{\tau }\cdot \vec{n}\right)\vec{n}$$ (3)

Eq. 3 was used to calculate WSS as follows: $\left|{\vec{\tau }}_{shear}\right|=\sqrt{\vec{\tau }\cdot \vec{\tau }-{\left(\vec{\tau }\cdot \vec{n}\right)}^{2}}$ . TAWSS was used to evaluate the total shear stress exerted on the wall throughout the cardiac cycle, which could be obtained from the following equation:

$$\mathrm{TAWSS}=\frac{1}{T}\int_{0}^{T}\left|{\vec{\tau }}_{shear}\right|\cdot dt$$ (4)

where T is one cardiac cycle.

The cyclic change in WSS was expressed by OSI in the range of 0–0.5 (Tan et al., 2009). The OSI characterized the changes in the intensity and direction as well as the degree of turbulence in the blood flow, using the following equation (Taylor et al., 1999):

$$OSI=\frac{1}{2}\left[1-\frac{\left|\frac{1}{T}\int_{0}^{T}{\vec{\tau }}_{shear}\cdot dt\right|}{\frac{1}{T}\int_{0}^{T}|{\vec{\tau }}_{shear}|\cdot dt}\right]$$ (5)

## Results

### Blood flow based on the four boundary conditions

At the primary entry (shown in Figure 1A) on M-I, there was a fast (>1 m/s) flow and vortex before TEVAR in Figure 2A. Herein, we selected three systolic time points: acceleration phase (t1), peak systolic (t2), and deceleration phase (t3). The blood flow in the FL was characterized by recirculation and stagnation, especially in the area near the top of the FL, which promotes thrombus formation (Menichini et al., 2016). At the systolic peak (t2), the mean flow velocity in the TL in the region below the stent-graft after TEVAR was increased compared to that before TEVAR; the velocity was reduced to ∼0.1 m/s in the FL. The CA also showed locally high flow rates, indicating possible stenosis. The velocity at the cross-section of the blood vessel was used as Setting 1 for CFD calculations. Figure 2 depicts the instantaneous streamlines of 4D flow MRI at three systolic time-points (t1–-t3) of M-I before thoracic endovascular aortic repair and after TEVAR. The flow rate of M-I in the TL was higher than that in the FL, and the velocity increased further along the descending aorta due to the narrowing of the TL caused by enlarged FL. In M-I, only a slight change was observed in the arterial flow (CA/SMA/RRA) after TEVAR, but LRA peak systolic velocity was increased two-fold as that preoperatively (Supplementary Figure S2). In the case of M-II, except for the CA, the flow rates of the other three branch arteries (SMA/RRA/LRA) were increased slightly, and the flow rates of the LRA and RRA were similar.

**FIGURE 3:**
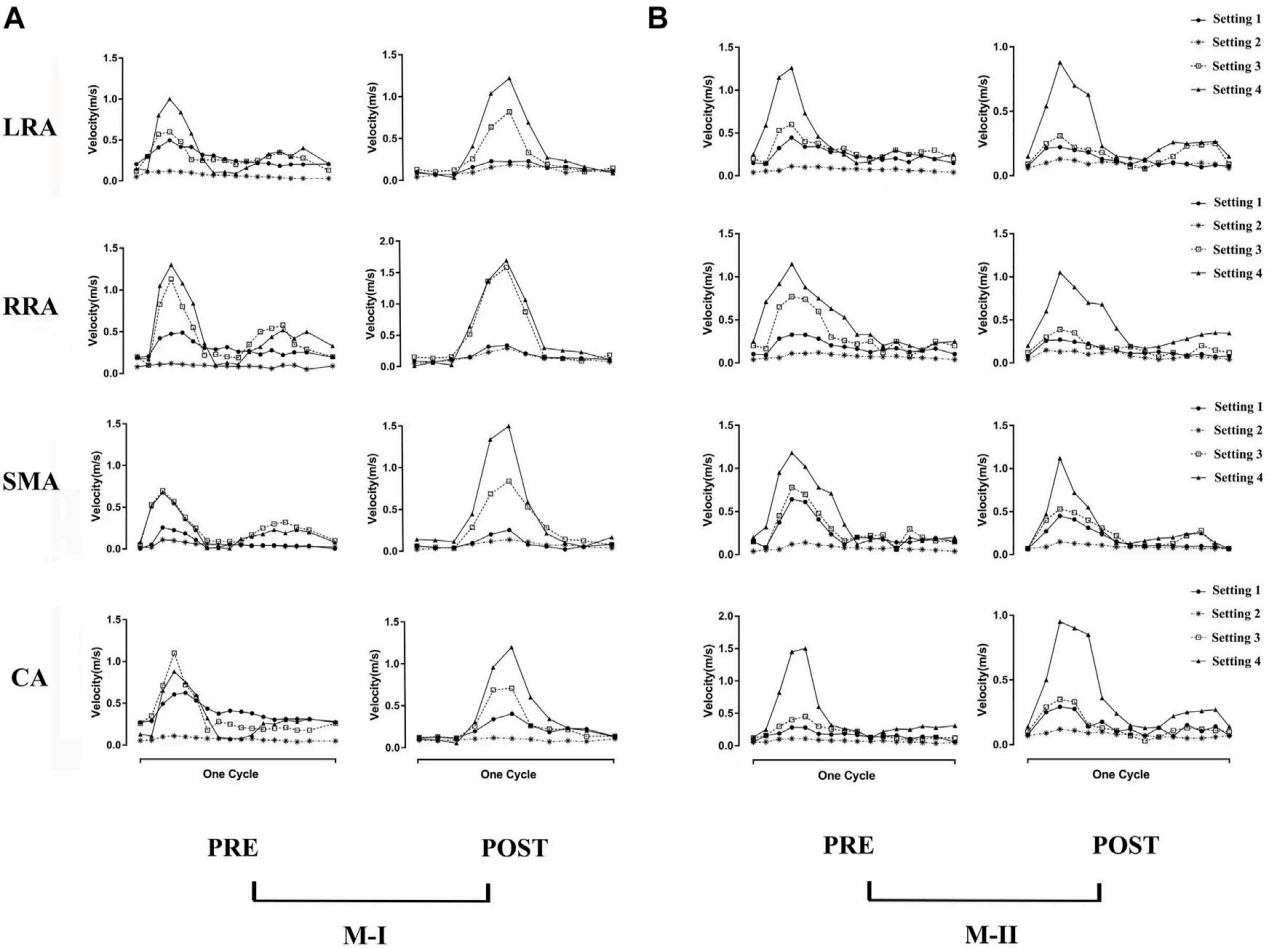
**(A)** shows the preoperative (pre) and postoperative (post) transient velocity of the left renal artery (LRA), right renal artery (RRA), superior mesenteric artery (SMA), and celiac artery (CA) on M-I in one cardiac cycle, and **(B)** on M-II.

Figure 3 shows the instantaneous flow velocities of the four branches of the abdominal artery in the four calculation results of M-I and M-II before and after the surgery. We select the section at the same position as the data collected in Supplementary Figure S2 to obtain the mean velocity value of each outlet, corresponding to the same time-point acquired in the 4D flow MRI data. Among all the results, the mean flow velocity of four abdominal outlets with Setting 3 and Setting 4 was much higher than Setting 1, and the trend for pre-TEVAR and post-TEVAR observed for them are agreeable. In the acceleration and deceleration phases, these two boundary conditions excessively distributed the blood flow from the abdominal aorta. However, the velocity of the Setting 2 result was lower than that of the Setting 1 results and was almost zero. Also, no difference was observed between the preoperative and postoperative values or between M-I and M-II in the Setting 2 results. Moreover, the Setting 3 results of the four branch arteries in M-I and M-II before and after the surgery were similar to the Setting 1 results.

**FIGURE 4:**
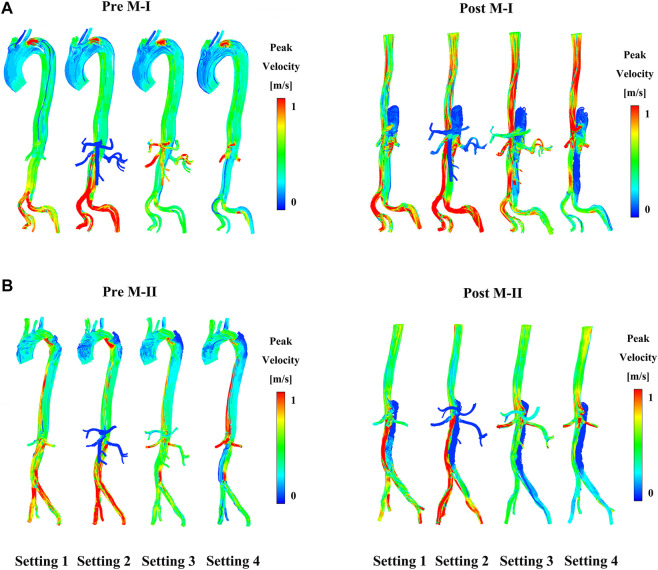
**(A)** Shows the preoperative (pre) and postoperative (post) peak-systolic instantaneous velocity streamlines under different boundary conditions on M-I and **(B)** on M-II.

Compared to before TEVAR, the LRA of M-I was affected by Tear 1 perfusion after TEVAR, which resulted in a higher velocity and a significant flow rate of the LRA than in the FL flow (Supplementary Figure S3). In addition, no difference was detected between the LRA and RRA in M-II before or after TEVAR; however, the velocity increased after TEVAR. Figure 4A shows the peak flow velocity of the abdominal aorta on M-I before and after TEVAR; Figure 4B M-II. The low flow rate of the branch arteries in the Setting 2 post-results showed a high flow velocity in the part of the artery below the abdominal branch arteries; it was about 25% greater than the Setting 1 in the FL on M-I (Supplementary Figure S4), while the Setting 3 and Setting 4 post-results were 20 and 23% lower than Setting 1. The calculations did not find any marked differences in the flow patterns in the ascending aorta and the aortic arch. But, setting 2 results were lower after TEVAR than before compared with setting 1, which refers to the part below the abdominal branches of M-II (planes 10–17). In addition, the comparison of all setting result of M-II patient with the original MRI data revealed that the various boundary conditions had no effect on the velocity distribution at timepoint t2 in the FL of M-II compared to M-I, which could be attributed to the fact that the branch arteries of the abdominal aorta were not involved in the FL of M-II.

### Hemodynamic parameters in the four boundary conditions

**FIGURE 5:**
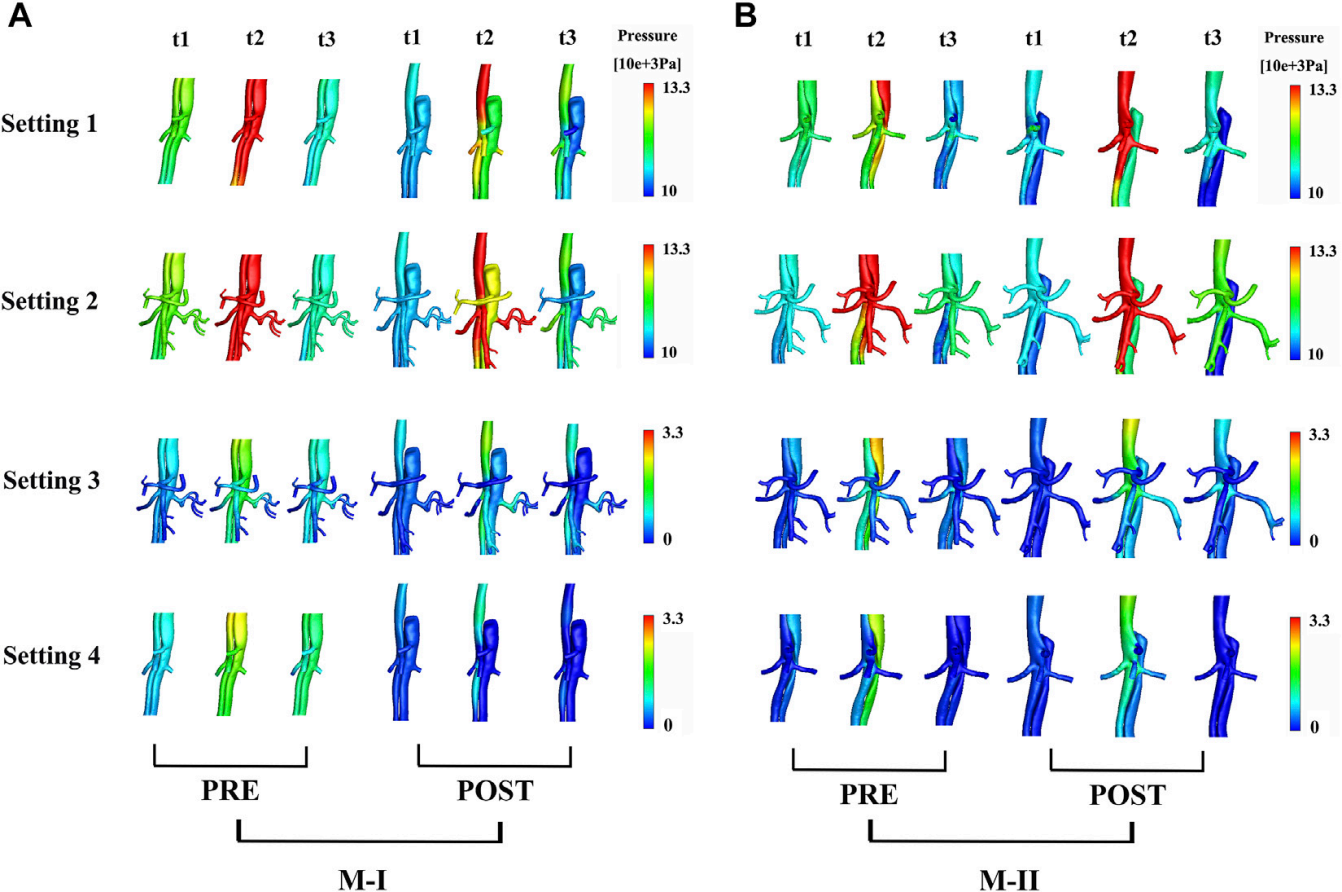
**(A)** shows the preoperative (pre) and postoperative (post) pressure distributions under different boundary conditions at three systolic time points (t1–t3) of M-I and **(B)** M-II.

Figure 5A shows the pressure distribution near the branches of the abdominal aorta at three different time points on M-I before and after TEVAR, and Figure 5B M-II. The maximum pressure at the peak systole (t2) reduced after TEVAR and decreased gradually along the aorta, with improvements in TL remodeling. After the operation, the fluid pressure at the wall of the TL in all cases was lower than that before TEVAR. At the peak of systole, the wall pressure difference between TL and FL in the Setting 1 results was maximal, but the zero pressure difference was minimal. In addition to the Setting 4 results, the wall pressure results of LRA at time points t2 and t3 were higher than those of FL in M-I but similar to those in M-II, which is agreeable in them after TEVAR compared with before. Tear-1 in M-I served as a bridge between the TL and FL to transport the flow and create a pressure gradient. These results indicated that the wall pressure of FL in M-II was more uniform than that in M-I, while the LRA and RRA in M-II were similar.

**FIGURE 6:**
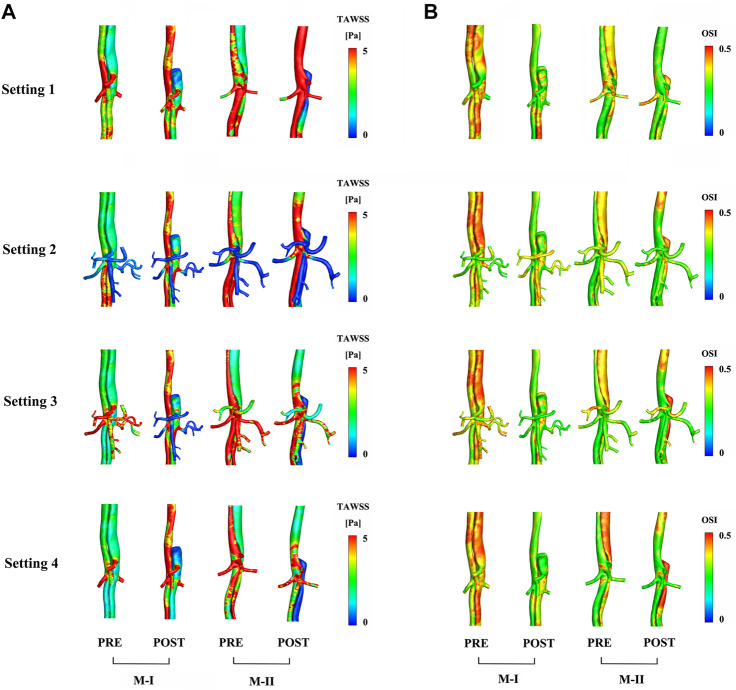
Comparison of **(A)** time-averaged wall shear stress and **(B)** oscillating shear index distribution between preoperative (pre) and postoperative (post) results.

In the CTA image, the LRA encompassed the FL of M-I, but not M-II, before and after the surgery. Also, a shadow (the area enclosed by the pink box) was observed in the left kidney in M-I after TEVAR, but not in M-II. This finding might indicate renal infarction in the shaded area, although in-depth investigations are essential (Supplementary Figure S5). The local infarction in the left kidney in M-I could be ascribed to the low velocity of LRA or the use of a large amount of contrast agent within a short period during the operation, which weakens renal functioning. Figure 6A presents the distributions in TAWSS at three time points, and Figure 6B OSI. The TAWSS values were higher in the TL than the extremely low values in the FL. The blood flow velocity in the narrowed part of the TL increased after TEVAR, which in turn increased the TAWSS, while the results of resistance, the Setting 3 and Setting 4 on the M-II did not exhibit a similar phenomenon. In M-I, the TAWSS in the area directly opposite to Tear-1 was increased by impingement of the jet through the tear. Initially, the FL had more areas with high OSI than the TL; however, only a few areas with high OSI were detected after than before TEVAR. The difference in TAWSS distribution was noted in the descending aorta among the varying boundary conditions and the trend for pre- and post-TEVAR are agreeable. The absolute percentage differences of TBAD between surface area ratio of TAWSS and surface area ratio of OSI with respect to the Setting 1 results were up to 23% $\left(=\frac{surface_{TAWSS\leq 4dynes\cdot cm^{-2}}}{surface\;area}\times 100\%\right)$ and 8% $\left(=\frac{surface_{OSI\geq 0.15}}{surface\;area}\times 100\%\right)$ in Setting 2 results, 35% and 12% in Setting 3 results, and 43% and 15% in Setting 4 results, respectively (Huo et al., 2013; Huang et al., 2018).

## Discussion

Previous studies have used constant pressure (Gao et al., 2006; Tang et al., 2012), outflow (Vignon-Clementel et al., 2006), and resistance boundary conditions (Yin et al., 2018) of the abdominal aortic branches in the calculation of a large number of cases to find clinical significance and regularity. Different flow-field states have been obtained in these studies based on various arterial shapes reconstructed using CTA. However, the setting of idealized boundary conditions in the TBAD calculation cannot just use the primary entry as the entrance to another channel (FL), ignoring the fact that the low blood flow velocity in FL is due to a tear in the inner layer of the aortic wall. Although the pressure guidewire can yield pressure data, the collection of invasive data is not conducive to the treatment of acute diseases and is not cost-effective. In the present study, we reproduced the flow patterns of the proximal aortic arch and the abdominal aorta based on the MRI data to analyze the hemodynamics before and after TEVAR. Boundary conditions are crucial for the distribution and directly affect the pressure and velocity distribution of the flow field. The flow field of the MRI-CFD results was more consistent with the initial MRI data than other optimized boundary condition results.

Previous studies (Morbiducci et al., 2013; Pirola et al., 2018) have shown that idealized velocity curves are not suitable while focusing on the ascending aorta and aortic arch, while the flow field of the descending aorta was not affected markedly by changes in the inlet velocity profile. Dillon-Murphy et al. (Dillon-Murphy et al., 2016) and Pirola et al. (Pirola et al., 2019) did not compare the CFD results of the descending aorta below the splanchnic branch arteries with real MRI data. On the other hand, Pirola et al. used the differences between the upstream and downstream average aortic flow from the 4D flow MRI data to collect data on the flow in the visceral arteries as a boundary condition for CFD hemodynamic assessment (Pirola et al., 2019). However, the definition (Pirola et al., 2017) of the flow split based on the cross-sectional areas does not apply to the visceral arteries in the abdomen due to the presence of vortex and reflux in the FL, especially when the FL involves the branches of the descending aorta.

Herein, we compared the three idealized boundary condition results with patient-specific CFD results and observed that the Setting 1 obtained from 4D flow MRI data could analyze the flow in the TL, FL, and branch arteries of the abdomen before and after TEVAR. Owing to the formation of thrombus in the stent graft after TEVAR, the flow into the FL was reduced, affecting the distal organ and limb perfused by FL (Patton and Clements, 2013). Furthermore, the decreased blood flow in the FL increases the velocity in the TL, with an increase in flow eccentricity and vorticity (Wentzel et al., 2005; Morbiducci et al., 2007; Pirola et al., 2019). The results based on the Setting 4 showed that the abdominal branch arteries distributed the blood flow to the aorta, especially in M-I patients with FL involving the splanchnic arteries.

In actual blood flow, the Setting 3 and Setting 4 could not characterize the variations in the resistance and compliance of the downstream of the branch arteries with the blood flow movement; hence, the Setting 3 would increase the errors in results. The fluid total stress is an essential factor in the development of TBAD and FL rupture (Xu et al., 2017) because the impact of hypertension on the blood flow has a continuous effect on the blood vessel wall, which in turn damages the vessel wall and alters the stiffness and elasticity (Shi et al., 2016). The pressure difference between the TL and FL, based on the Setting 2, Setting 3, and Setting 4, does not increase the prognostic accuracy.

The hemodynamic characteristics are related to the complications and outcomes of TEVAR (Osswald et al., 2017). Incorporating the boundary conditions of the abdominal aortic branches based on MRI data would be valuable while using hemodynamic parameters to guide clinical, surgical planning, especially for the postoperative evaluation of the descending aorta. The effect of the application of varied boundary conditions is noticeable in the values of the TAWSS that exhibited differences in the hot-spot values and their distribution. In detail, the application of Setting 3 and Setting 4 decreases the TAWSS values to lower than other boundary conditions, with less difference in the OSI. These results demonstrated that different boundary conditions at the vessels outlet sections influence flow separation at branching. The TAWSS was lower in most areas of the FL than that before TEVAR, and this was associated with the formation and propagation of the thrombus (Dake et al., 1999; Borghi et al., 2008; Menichini et al., 2016). The high wall pressure and TAWSS value close to the LRA in M-I might lead to aneurysm formation, FL enlargement, and production of new tears (Osswald et al., 2017). The OSI distribution increased in most areas of the FL, indicating that the disordered flow streamlines in the FL were more turbulent than those in the TL and were severely subjected to shear stress in the alternate direction.

## Limitations

Nevertheless, the present study has some limitations. First, the pre- and post- 4D flow MRI data for all branch arteries mentioned in this study were limited and hence, we selected two typical patients as the study subjects. Due to the lack of measurements of wall thickness and mechanical properties related to simulations, the assumption of a rigid wall was used in the calculation model of TBAD. Second, the method based on MRI data could be used for FSI simulations because some studies demonstrated that the effect of rigid wall assumption on FL flow could be negligible (Alimohammadi et al., 2015; Selene et al., 2019). Although the present study did not focus on uncertainty quantification, the problem related to the characterization of the effects of the uncertainties in the inlet flow-rate waveform and in the outlet boundary conditions on hemodynamics and stresses should be investigated. Additional studies are required in the future to validate the pressure difference between the TL and FL based on 4D flow MRI. Third, local disruptions could occur where the target curvature is large, leading to turbulence in extreme conditions. This pilot study is based on clinical applicability, and follow-up studies should focus on direct numerical simulation.

## Conclusion

In this study, 4D flow MRI data were used to derive the patient-specific boundary conditions for CFD analysis in the two types (FL with and without the involvement of visceral arteries in the abdomen) of TBAD. Also, the hemodynamic parameters of the two types of TBAD were discussed based on various boundary conditions. The results showed that the boundary conditions had an impact on the calculations of the abdominal flow field and wall pressure. Our model of aortic dissection incorporated the boundary conditions of the abdominal branch arteries based on MRI data into numerical simulations, which might closely represent the flow field state of the descending aorta more than the idealized boundary conditions. This phenomenon might be valuable in future clinical data analysis of CFD calculations.

## Data Availability

The original contributions presented in the study are included in the article/Supplementary Material, further inquiries can be directed to the corresponding authors.
