# Supplementary material for: Influence of MRI-based boundary conditions on type B aortic dissection simulations in false lumen with or without abdominal aorta involvement
Source: Front Physiol. 2022 Sep 7;13:977275. doi: 10.3389/fphys.2022.977275 (PMC9490059; doi:10.3389/fphys.2022.977275)
Supplement: Supplementary file 1 [file DataSheet1.ZIP › Supplement File 1 R3.docx]

**Resistance outlet boundary condition**

Resistance was defined as a constant relationship between average pressure and flow, P = QR (Westerhof et al., 2009). We assume that the pressure in the downstream region was constant over the cross-sectional area of the inlet boundary. According to the law of diameter flow (Huo and Kassab 2009, Huo and Kassab 2012), the blood flow at outlet i $\left( Q_{mean} \right)_{i}$ (the time average of the cardiac cycle) was estimated as:

$\left( Q_{mean} \right)_{i}=Q_{total}\times\frac{D_{i}^{7/3}}{\sum_{j=1}^{N} D_{j}^{7/3}}$ (6)

$\left( V_{mean} \right)_{i}=\frac{\left( Q_{mean} \right)_{i}}{\frac{\pi}{4}\times D_{i}^{2}}$ (7)

where N is the total number of outlets of arterial trees reconstructed from CTA images and $\left( V_{mean} \right)_{i}$ (time-averaged over a cardiac cycle) is the outlet flow velocity. A steady-state flow simulation was carried out with the inlet pressure of $\left( P_{mean}-P_{0} \right)$ and outlet flow velocity, where $P_{mean}$ is the time-averaged aortic pressure over a cardiac cycle and the zero-flow pressure, $P_{0}$, was set to 51 mmHg (Dole et al., 1984). The pressure at outlet i, $\left( P_{mean} \right)_{i}$ (time-averaged over a cardiac cycle), was obtained from the steady-state computation. The resistance at each outlet of arterial tree, $R_{outlet}$, can be determined as:

$R_{outlet}=\frac{\left( P_{mean} \right)_{i}-P_{0}}{\left( Q_{mean} \right)_{i}}$ (8)

In the transient flow simulation, $R_{outlet}$ was assumed to be constant. Hence, we obtained the following equation as:

$V_{outlet}=\frac{P_{outlet}-P_{0}}{R_{outlet}\times\frac{\pi}{4}\times D_{outlet}^{2}}$ (9)

where P_outlet_ and V_outlet_ refer to the transient pressure and flow velocity, respectively, at each outlet of arterial tree. Equation [7] was used as the transient resistance boundary condition with V_outlet_ and P_outlet_ being the transient variables in a cardiac cycle.

**Hemodynamic Parameters:**

The shear component of $\vec{\tau}$ was expressed as follows:

$\tau=\left[ \begin{matrix} \tau_{11} & \tau_{12} & \tau_{13} \\ \tau_{21} & \tau_{22} & \tau_{23} \\ \tau_{31} & \tau_{31} & \tau_{33} \end{matrix} \right]=2\mu D=\mu\left[ \begin{matrix} 2\frac{\partial u}{\partial x} & \frac{\partial u}{\partial y}+\frac{\partial v}{\partial x} & \frac{\partial u}{\partial z}+\frac{\partial w}{\partial x} \\ \frac{\partial u}{\partial y}+\frac{\partial v}{\partial x} & 2\frac{\partial v}{\partial y} & \frac{\partial v}{\partial z}+\frac{\partial w}{\partial y} \\ \frac{\partial u}{\partial z}+\frac{\partial w}{\partial x} & \frac{\partial v}{\partial z}+\frac{\partial w}{\partial y} & 2\frac{\partial w}{\partial z} \end{matrix} \right]$ (10)

Where $D=0.5\cdot\left[ \left( \nabla\vec{V} \right)+\left( \nabla\vec{V} \right)^{T} \right]$ is the shear rate tensor. The stress on the wall, its normal component, and its two tangential components can be written as, respectively:

$$\vec{\tau}=\tau\cdot\vec{n}$$

$\tau_{n}=\vec{n}\cdot\tau\cdot\vec{n}$ (11)

$\tau_{t1}=\vec{t_{1}}\cdot\tau\cdot\vec{n}$ and $\tau_{t2}=\vec{t_{2}}\cdot\tau\cdot\vec{n}$

where $\vec{n}$, $\vec{t_{1}}$, and $\vec{t_{2}}$ are the unit vector in the normal and two tangential directions, respectively. The shear component of $\vec{\tau}$ has the vector form:

$\vec{\tau}_{shear}=\vec{\tau}-\left( \vec{\tau}\cdot\vec{n} \right)\vec{n}$ (12)
