# Supplementary figures and images for: Influence of MRI-based boundary conditions on type B aortic dissection simulations in false lumen with or without abdominal aorta involvement
Source: Front Physiol. 2022 Sep 7;13:977275. doi: 10.3389/fphys.2022.977275 (PMC9490059; doi:10.3389/fphys.2022.977275)

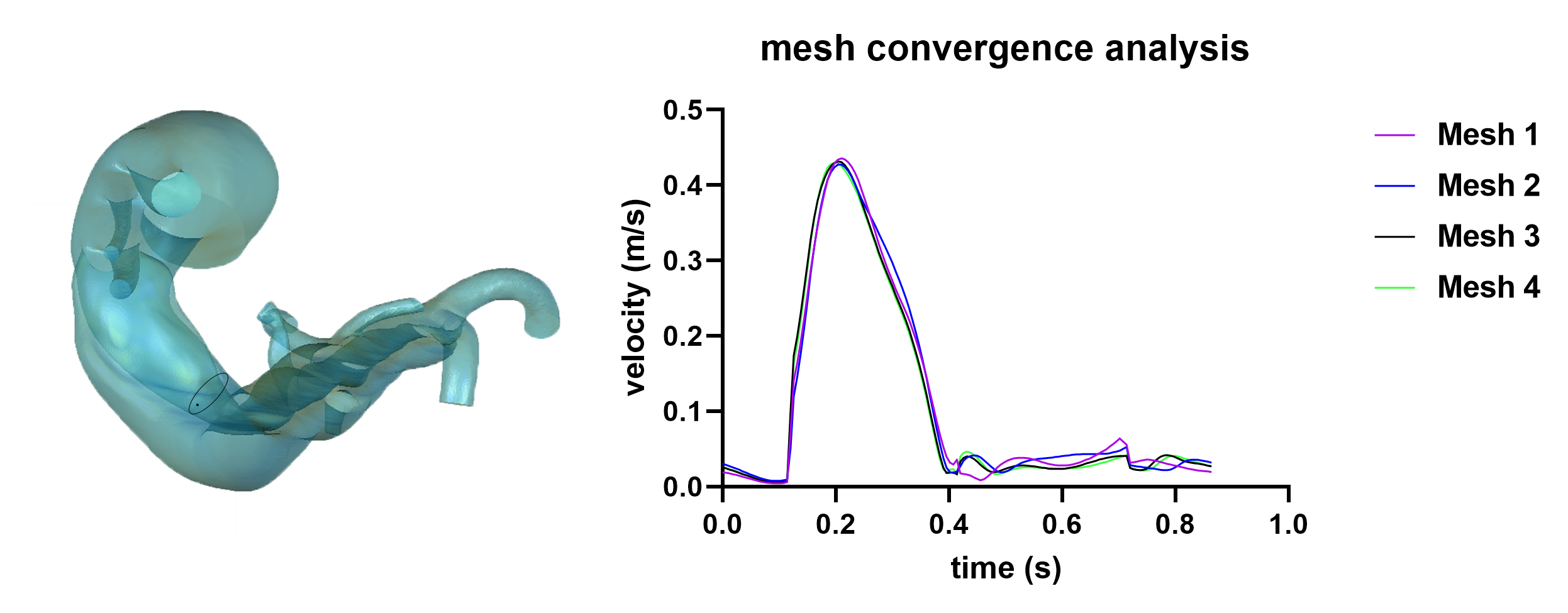

Supplement: Supplementary file 1 [file DataSheet1.ZIP › Figure 1 Supplementary.tif]

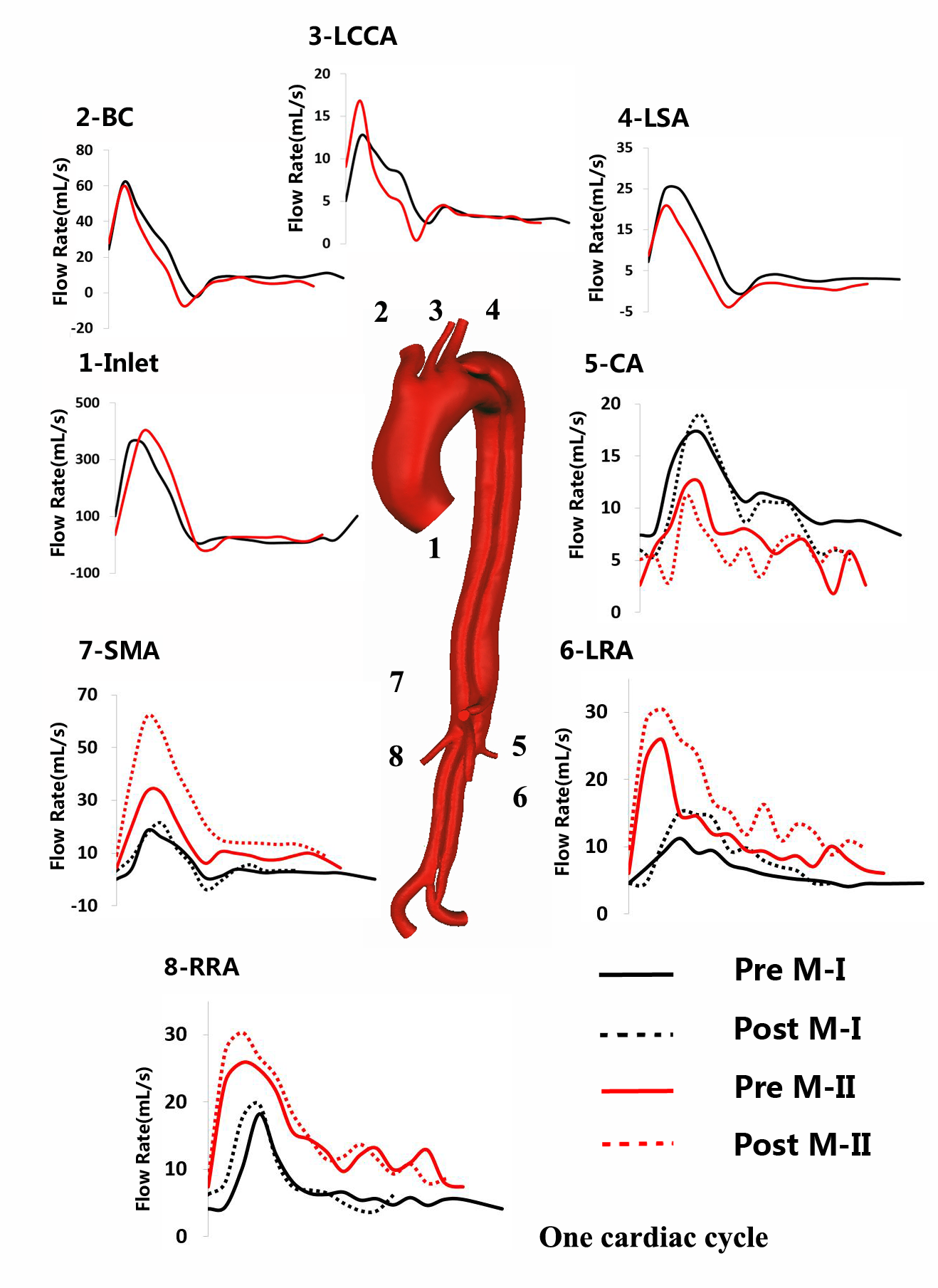

Supplement: Supplementary file 1 [file DataSheet1.ZIP › Figure 2 Supplementary.tif]

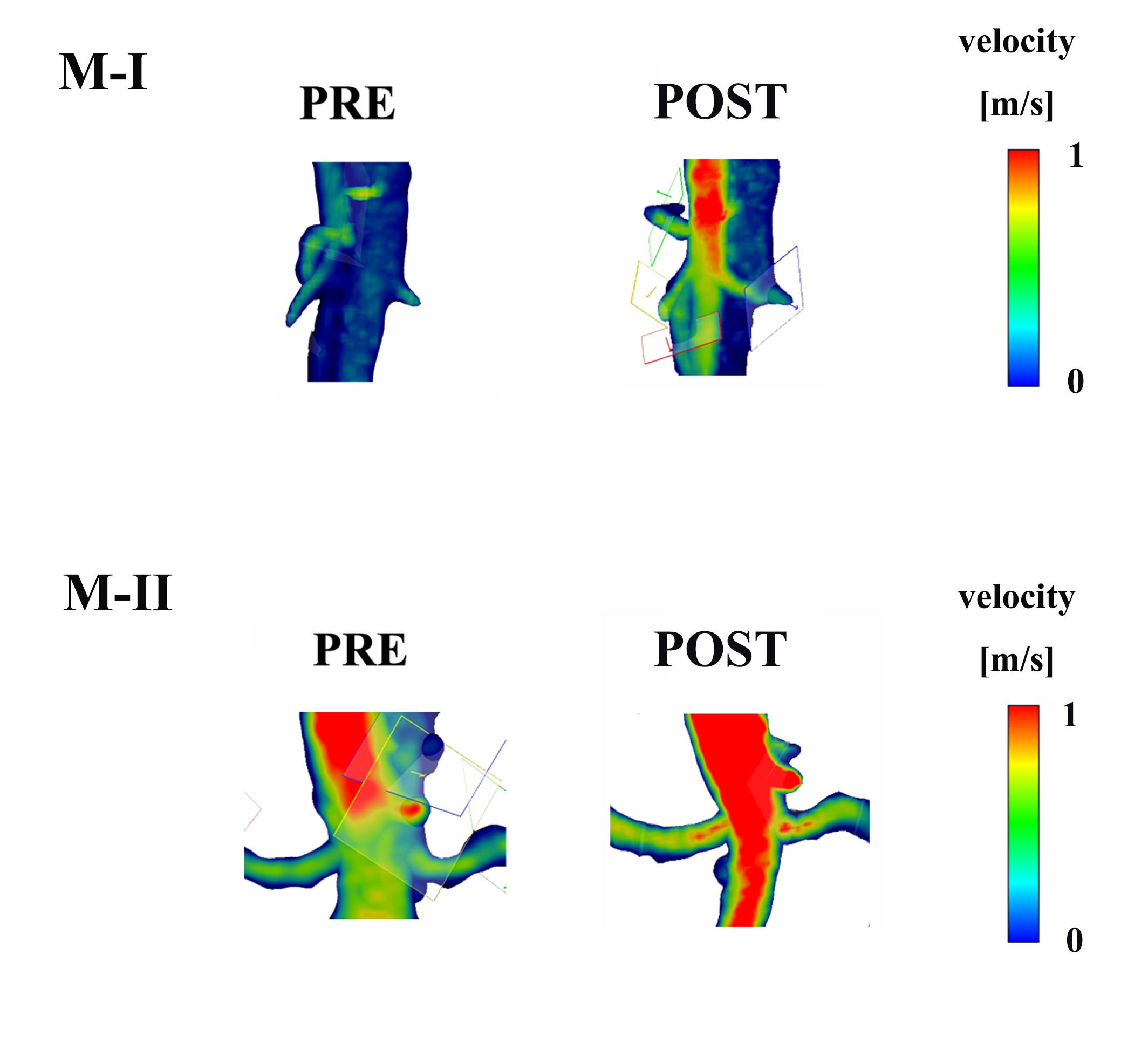

Supplement: Supplementary file 1 [file DataSheet1.ZIP › Figure 3 Supplementary.tif]

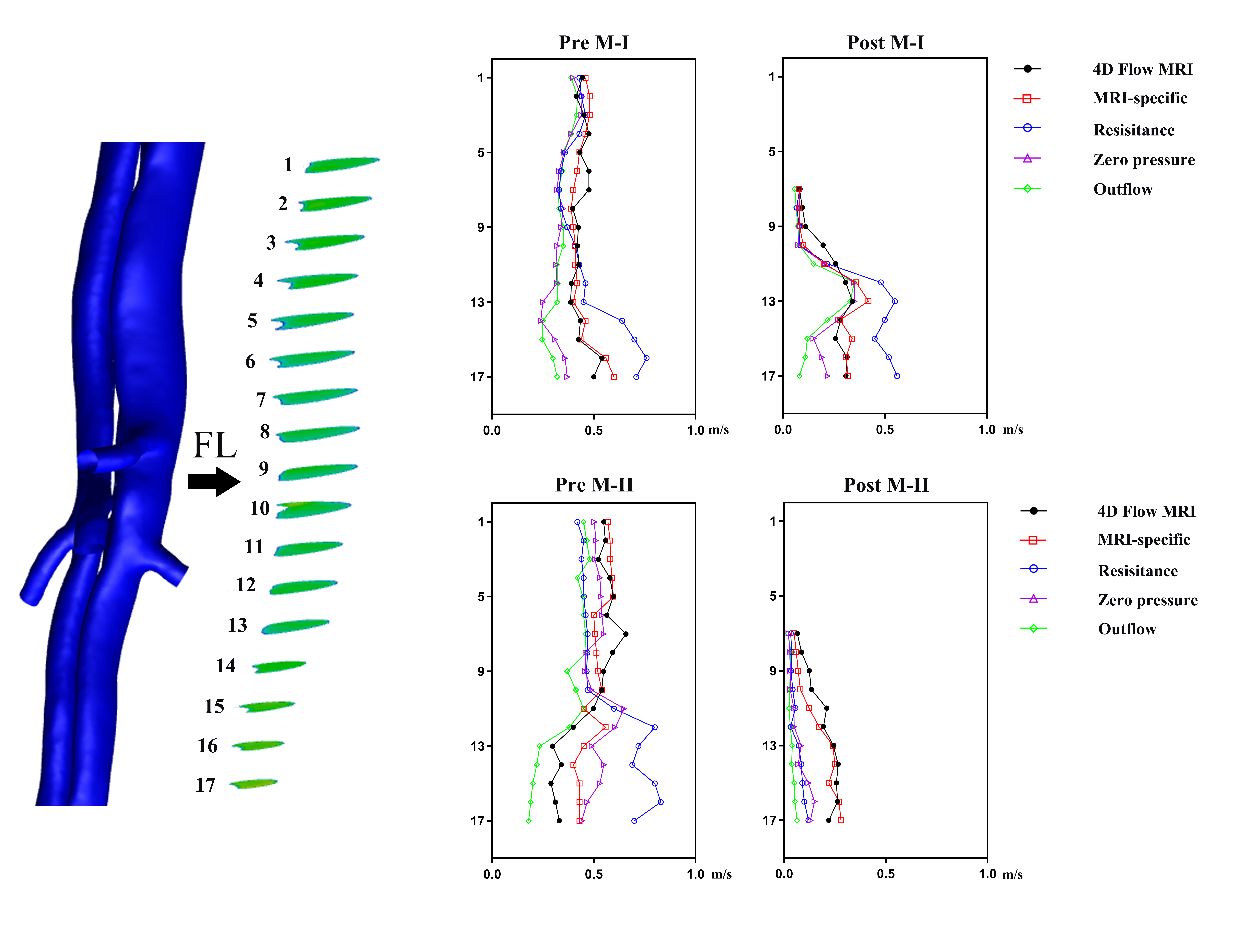

Supplement: Supplementary file 1 [file DataSheet1.ZIP › Figure 4 Supplementary.tif]

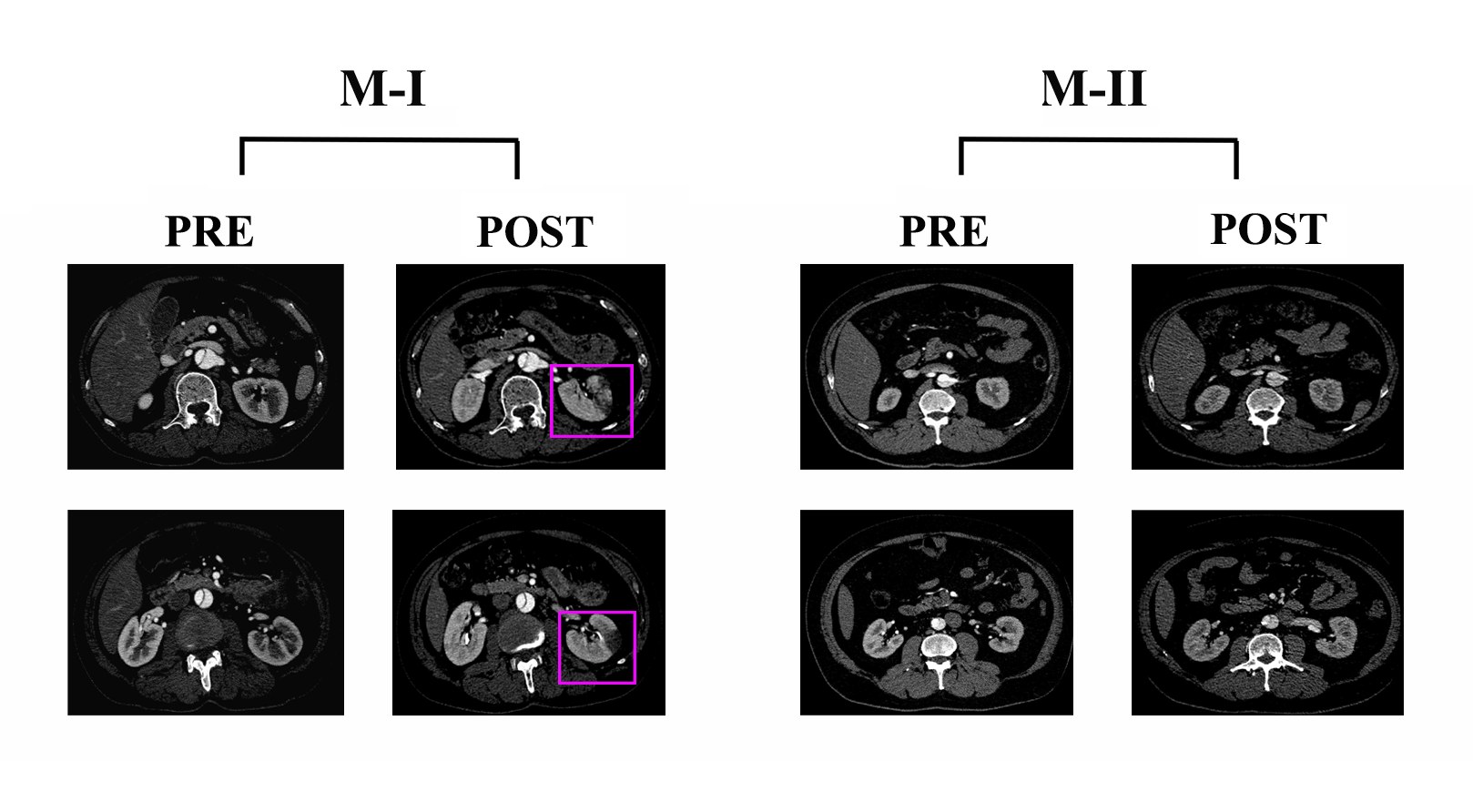

Supplement: Supplementary file 1 [file DataSheet1.ZIP › Figure 5 Supplementary.tif]
